# American Sign Language Translation Using Wearable Inertial and Electromyography Sensors for Tracking Hand Movements and Facial Expressions

**DOI:** 10.3389/fnins.2022.962141

**Published:** 2022-07-19

**Authors:** Yutong Gu, Chao Zheng, Masahiro Todoh, Fusheng Zha

**Affiliations:** ^1^Graduate School of Engineering, Hokkaido University, Sapporo, Japan; ^2^Wuhan Second Ship Design and Research Institute, China State Shipbuilding Corporation Limited, Wuhan, China; ^3^Faculty of Engineering, Hokkaido University, Sapporo, Japan; ^4^State Key Laboratory of Robotics and System, Harbin Institute of Technology, Harbin, China

**Keywords:** American sign language, inertial measurement units, electromyography, long short-term memory, transformer

## Abstract

A sign language translation system can break the communication barrier between hearing-impaired people and others. In this paper, a novel American sign language (ASL) translation method based on wearable sensors was proposed. We leveraged inertial sensors to capture signs and surface electromyography (EMG) sensors to detect facial expressions. We applied a convolutional neural network (CNN) to extract features from input signals. Then, long short-term memory (LSTM) and transformer models were exploited to achieve end-to-end translation from input signals to text sentences. We evaluated two models on 40 ASL sentences strictly following the rules of grammar. Word error rate (WER) and sentence error rate (SER) are utilized as the evaluation standard. The LSTM model can translate sentences in the testing dataset with a 7.74% WER and 9.17% SER. The transformer model performs much better by achieving a 4.22% WER and 4.72% SER. The encouraging results indicate that both models are suitable for sign language translation with high accuracy. With complete motion capture sensors and facial expression recognition methods, the sign language translation system has the potential to recognize more sentences.

## Introduction

Sign language is the main communication method among hearing-impaired people. According to the World Federation of the Deaf, there are 70 million deaf people around the world using sign language in their daily life. As a kind of natural language, sign language has not become a mainstream Research Topic in natural language processing (NLP), although the machine translation of spoken or written language is highly accurate today. However, the research on machine translation with deep learning models provides development direction and innovative methods for sign language translation tasks. To further research on end-to-end translation, it is necessary to consider the application of deep learning models (Bing et al., 2021a,b,c, 2022).

Previous works about sign language translation mainly fall into two categories: vision-based and wearable sensor-based methods. Vision-based methods exploit cameras to capture features of the hands (Koller et al., 2015; Sun et al., 2015; Fang et al., 2017). One most commonly used dataset is RWTH-PHOENIX-Weather (Koller et al., 2015), which contains 3 years' sign language interpretation of daily news and weather forecast from a German public TV-station. With this dataset, a machine translation model with an encoder-decoder structure was built that included both long short-term memory (LSTM) and connectionist temporal classification (CTC) as the decoder (Pu et al., 2019). The transformer-based architecture was applied to make the model trainable in an end-to-end manner (Camgoz et al., 2020). A continuous sign recognition framework named Hierarchical Attention Network with Latent Space (LS-HAN) was proposed (Huang et al., 2018). Another well-known dataset is Chinese Sign Language (CSL) (Zhou et al., 2019). This dataset containing 100 continuous Chinese sign language sentences was collected by the Kinect device. A novel architecture with cross-modality augmentation reached state-of-the-art translation accuracy (Pu et al., 2020).

In wearable sensor-based research, devices, such as data gloves, wristwatches, or armbands, are the mainstream for data collection (Cheng et al., 2015; Wei et al., 2016). Inertial data and surface electromyography (EMG) data were collected from forearms to detect hand/arm gestures (Wu et al., 2016). In total, 80 commonly used American sign language (ASL) signs were classified by a support vector machine classifier. An ASL translation system named MyoSign was presented using the MYO armband as a data collection device (Zhang et al., 2019). The end-to-end translation model consisted of convolutional neural network (CNN), LSTM, and CTC layers. In total, 100 sentences that comprised of 70 commonly used ASL words without considering sign language grammar were translated with more than 92% accuracy. Another work using the MYO armband collected data from 20 ASL sentences and treated it as a classification task (Tateno et al., 2020). The LSTM classifier could recognize these twenty motions with high accuracy among twenty participants. With one sample entropy-based feature set for both accelerometer and EMG, 60 isolated Greek Sign Language signs were recognized with an accuracy of 93% (Kosmidou and Hadjileontiadis, 2009). The combination of the MYO armband and Leap Motion camera was used to estimate continuous hand position (Quivira et al., 2018). Combining deep learning with principal component analysis (PCA), the grasp of a prosthetic hand was controlled (Li et al., 2018).

Sign languages are not exactly expressed with hands. It is also critical to catch facial expressions. For example, raising eyebrows means an open-ended question in ASL (Bragg et al., 2019). In video-based translation, it is easy to catch the movements of hands and face with a camera simultaneously. However, a few studies have considered facial expressions as important information. For wearable sensors, facial EMG data are widely used in emotional classification. Five different facial emotions were classified with 2-channel EMG sensors and a CNN classifier (Kehri and Awale, 2020). Emotion recognition with EMG measurements of the zygomaticus major and corrugator supercilii muscles was studied to identify happy, angry, and neutral faces (Kulke et al., 2020). In naturalistic studies, facial EMG signals can also be used to assess subjective emotional valence. Wearable devices with EMG electrodes were developed to record participants' facial changes while viewing emotional films (Sato et al., 2021). With the evaluation of facial EMG, emotional facial expressions in real-life social interactions were more evocative of reactions than experimental conditions (Hsu et al., 2020).

In summary, with deep learning models, both vision-based and wearable sensor-based methods can translate human movements into text sentences during sign language performance. The vision-based works tend to build and train the model on benchmarks with advanced algorithms and data augmentation. Wearable sensor-based topics always collect data by themselves due to different kinds of devices applied in experiments. With only EMG signals from forearms, limited sign language words or sentences can be recognized accurately. After adding the data from inertial measurement units (IMU), results are significantly improved (Zhang et al., 2019).

In our work, we have applied IMU signals from forearms and hands to translate 40 ASL sentences into texts following the grammar rules. To realize end-to-end translation, two kinds of encoder-decoder structured models in NLP are included: the LSTM-based model and transformer-based model. To acquire more information, facial expression data collected by EMG sensors are also regarded as a part of the input to translation models. The rest of the paper is organized as follows. We first collected IMU and EMG signals and did signal preprocessing. Then we presented LSTM and transformer models and trained the models with the dataset. The models were evaluated by the testing dataset, and the significance of the EMG signal was discussed. Finally, the discussion and conclusion of the paper were drawn.

## Materials and Methods

### ASL Specifics

American sign language is a kind of visual language expressed *via* a sequence of sign gestures. A sign consists of four main components, i.e., hand shape, movement, palm orientation, and location. In addition, facial expression can also be critical to express the signer's current mood. For example, raised eyebrow always indicates asking a question and a neutral face conveys a statement of fact. In addition to neutrality and questioning, positive and negative emotions are also considered in this research. In total, 40 commonly used sentences (listed in Table 1) with emotions positive, negative, questioning, and neutral were selected for recognition. These 40 sentences come from popular sign language videos on the Internet. The signers perform these sentences with obvious facial expressions.

**Table 1:** Forty commonly used American sign language sentences.

**Positive**	**Negative**	**Questioning**	**Neutral**
1. I'm happy!	11. Today I feel sad.	21. Are you deaf?	31. I'm fine.
2. Wow the steak is delicious!	12. I don't like cat.	22. Are you finish?	32. I'm busy.
3. Happy new year!	13. Why you are sad.	23. Are you alright?	33. I need help.
4. Merry Christmas!	14. I'm afraid of spider.	24. Do you want milk and cookies?	34. You like him.
5. Wow the dessert is delicious!	15. Running, growing up, I hate it.	25. Do you like ice-cream?	35. I go to church on Sunday.
6. Haha the commercial is funny!	16. I don't know where, sad.	26. Are you happy with studying history?	36. I'm a broke college student.
7. With you I'm happy!	17 My friend dislikes wrestling.	27. Do you come to church on Sunday?	37. I go to beach this summer.
8. Happy thanksgiving!	18. His wife dislikes cooking.	28. Do you also want fries?	38. We are hungry.
9. Happy mother's day!	19. I'm worried. they are angry.	29. Did you finish eating vegetable?	39. I go back home.
10. This year we are happy!	20. I feel annoyed.	30. Does this food have strawberry?	40. They enjoy eating hamburgers.

### Dataset Collection

**Figure 1:**
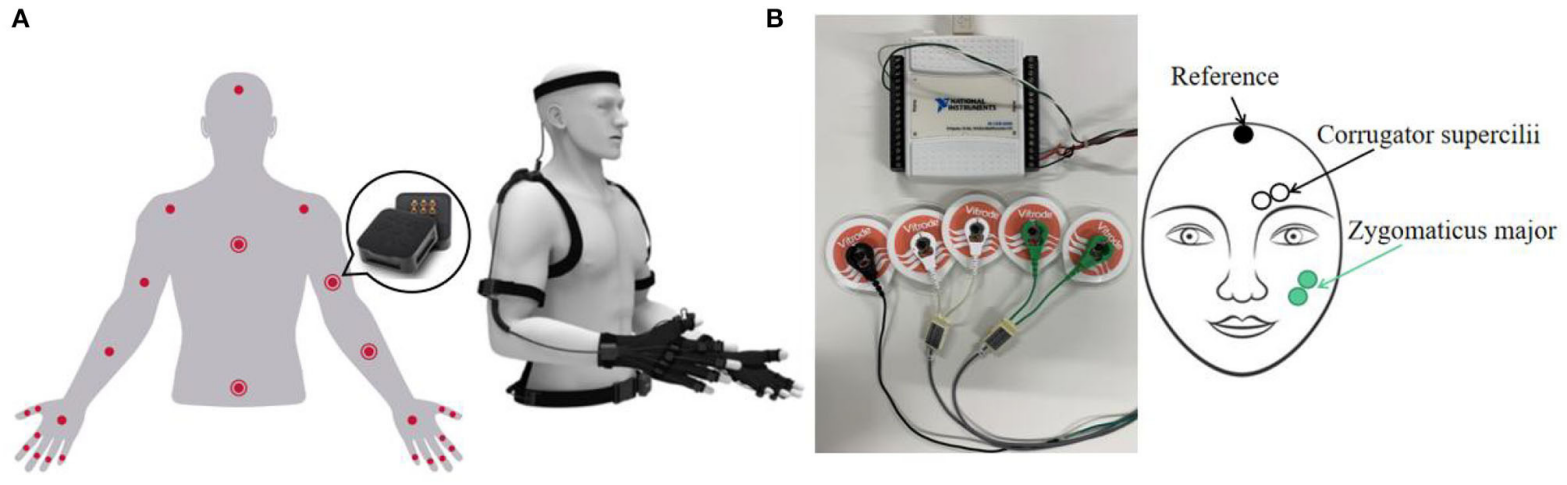
Devices for data collection: **(A)** perception Neuron motion capture system; **(B)** electromyography (EMG) signal acquisition system.

The movements of forearms and hands were obtained by the Perception Neuron Motion Capture System. As shown in Figure 1A, this system is based on wearable IMU sensors named “Neuron.” Each Neuron is composed of an accelerometer, gyroscope, and magnetometer. There are 25 Neurons for capturing upper body movements. The motion capture system needs to communicate with the Axis Neuron software. Axis Neuron can receive and process the data from all IMU sensors and export it into a *.bvh* format file. In this file, skeleton information and movement information of the whole process are recorded. We have only used the motion data, which record the rotation information of all joints of the human body. We have only focused on the data from hands and forearms' joints. The sampling rate was 60 Hz.

Electromyography (EMG) measures the electrical activity generated by the muscle. Figure 1B shows a 2-channel EMG signal acquisition system. The system mainly includes an NI data collector and differential electrodes. The NI USB-6008 provides eight single-ended analog inputs. Four single-ended analog inputs were used to form two differential channels. Another grounded channel was used as a reference. The electrode applied in this system was wet silver/silver chloride (Ag/AgCl) surface electrode. The useful information of EMG signals was mainly distributed in the frequency range of 0–500 Hz (De Luca et al., 2010). To meet the Nyquist sampling theorem, the sampling rate was chosen as 1 kHz.

In the experiment, EMG signals from zygomaticus major and corrugator supercilii areas and IMU signals from forearms and hands were collected. Three participants with the right hand as the dominant hand participated in data collection. The signers performed each sign language sentence with both hand movements and facial expressions. Participant 1 contributed the largest amount of data (1,600 samples). Participants 2 and 3 each contributed 400 samples. Finally, there were 60 samples for each sentence and 2,400 samples in total in the dataset.

### Data Pre-Processing

**Figure 2:**
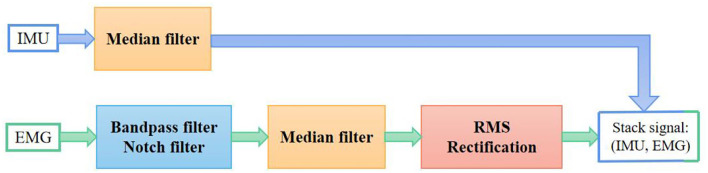
Signal preprocessing flowchart.

The *.bvh* data from the IMU motion capture system includes all the motion data of 59 bones. We had only focused on the data from hands and forearms. Finger spacing was fixed in Axis Neuron software; as a result, some channels maintained the same values throughout the experiment. We manually removed these channels that contained no useful information. Finally, only 38 channels were remained for the inertial data of forearms and hands. Since the IMU signals were sampled with a much lower sampling rate, we only used a median filter with a kernel size of 5 to make data smooth. The signal preprocessing flow is shown in Figure 2.

When compared to IMU, the EMG signal was much noisier and unstable. To maintain the performance of EMG features, the signal was band-passed and notch filtered to remove power-line interference and motion artifacts (Phinyomark et al., 2009). Then, a median filter was used to smooth the data. Rectification is a commonly applied approach to magnify the EMG features (Yang et al., 2016). The Root-Mean-Square rectification of signal *x(t)* is defined as


$$\begin{array}{l} EMG_{rect}(t)\;=\;\sqrt{\frac{1}{T}\int_{t-T}^{T}x^{2}(\tau )d\tau } \end{array}$$

**Figure 3:**
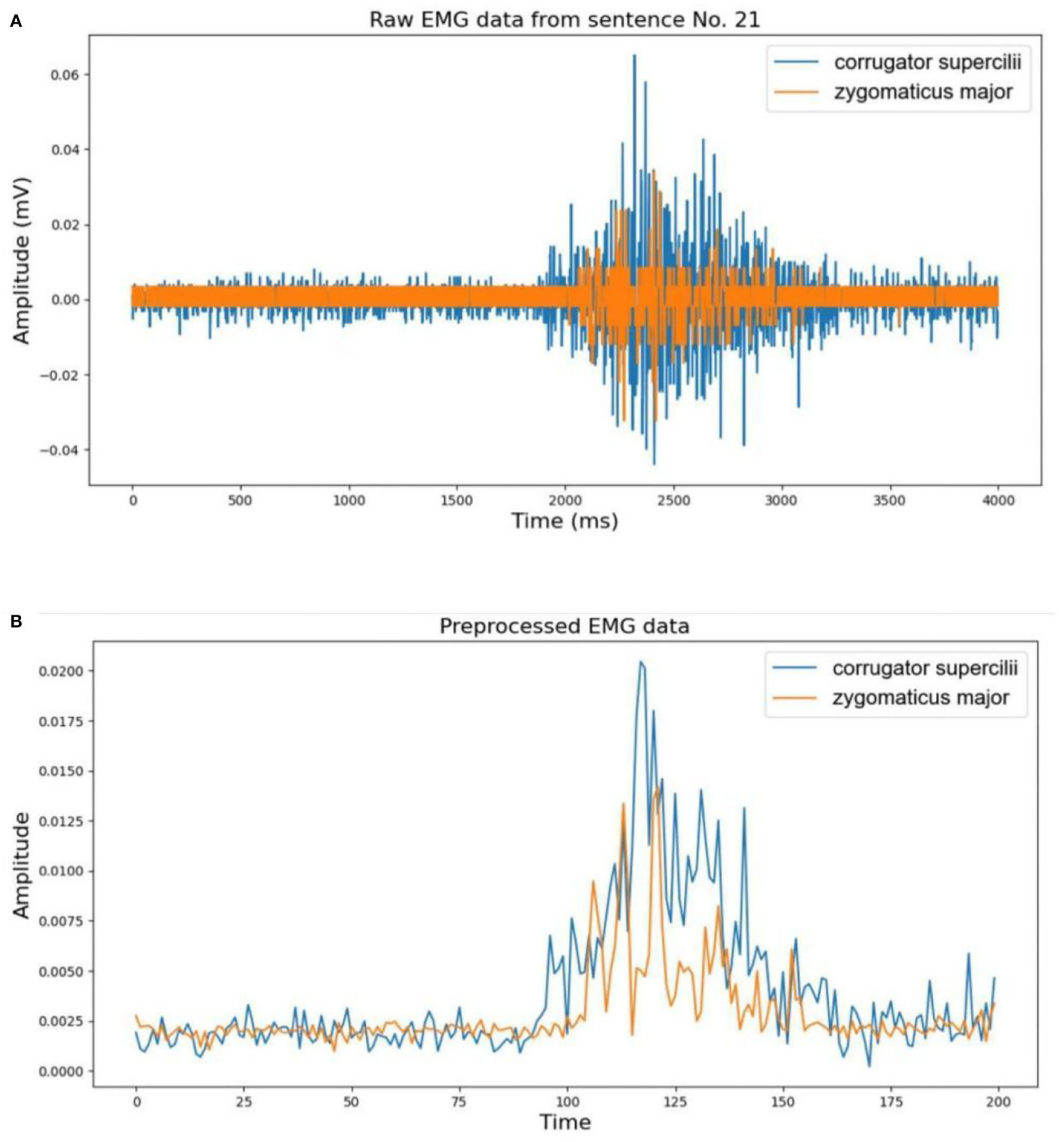
An example of electromyography (EMG) data preprocessing: **(A)** raw EMG data from sentence no. 21; **(B)** corresponding preprocessed EMG data.

Where *T* is the window size that controls the trade-off between smooth envelopes against transient variations of EMG signal. We set this value to be 0.02 s to avoid signal distortion and to keep approximately consistent in length with the IMU signal according to the sampling rates of the two devices. The lengths between EMG and the corresponding IMU signal may be different, so we resampled the EMG to the same length as IMU in the final step of preprocessing. An example of the EMG data from sentence no. 21 before and after preprocessing is shown in Figure 3.

### Facial Expressions Classifier

Convolutional neural network is an effective technique to solve signal and image classification problems. Based on shared-weights architecture, CNN eliminates effects from motion differences in amplitude and trajectory (LeCun et al., 1998). An emotional classifier using CNN as a feature extractor was proposed in this research.

**Figure 4:**
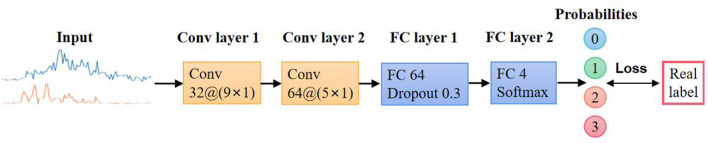
Facial expressions' classification model.

The CNN classifier mainly consists of four layers as shown in Figure 4. The first two layers are convolutional layers with 9 × 1 and 5 × 1 kernels, respectively. Since the input EMG signal contains two independent channels, to avoid any confusion, the convolutional kernels are both 1-D kernels. Batch normalization (Ioffe and Szegedy, 2015) was used for reducing internal covariate shift, and rectified linear unit (ReLU) was selected as the activation function. Max pooling was set to reduce the computational burden. The following layer is a fully connected layer with a dropout strategy to prevent overfitting (Srivastava et al., 2014). Finally, there is a fully connected layer with G-way softmax. G is the number of facial expressions to be recognized.

### Sign Language Translation Models

In the sign language dataset, the continuous signal stream for each sentence lasts for around 3–10 s. With the sliding window method, the long signal stream is segmented into a sequence of frames. Since the sampling rate of the motion capture system was 60 Hz, the window size we set was 600 ms (36 sample points) and the sliding size was 300 ms (18 sample points).

The label for collected EMG and IMU data was the corresponding text sentence. There were 40 sentences in the dataset that consisted of words and punctuation. We built a vocabulary at the word level and used the index of the word as the label. The vocabulary is shown in Table 2. Three kinds of special words were added to vocabulary: <BOS>, <EOS>, and <PAD> (indicated “begin of sentence,” “end of sentence,” and “padding”). We added <BOS> and <EOS> to the beginning and end of each sentence in the dataset and then padded the sentence to the same length with <PAD>. Finally, text sentences were changed into sequences of words' indices.

**Table 2:** The vocabulary for 40 American sign language (ASL) sentences.

!	,	.	?	Christmas	I	I'm	Sunday
A	Afraid	Alright	Also	And	Angry	Annoyed	Are
Back	Beach	Broke	Busy	Cat	Church	College	Come
Commercial	Cooking	Cookies	Day	Deaf	Delicious	Dessert	Did
Dislikes	Do	Does	Don't	Eating	Enjoy	Feel	Fine
Finish	Food	Friend	Fries	Funny	Go	Growing	Haha
Hamburgers	Happy	Hate	Have	Help	Him	His	History
Home	Hungry	Ice-cream	Is	It	Know	Like	Merry
Milk	Mother's	My	Need	New	Of	On	Running
Sad	Spider	Steak	Strawberry	Student	Studying	Summer	Thanksgiving
The	They	This	To	Today	Up	Vegetable	Want
We	Where	Why	Wife	With	Worried	Wow	Wrestling
Year	You	<BOS>	<EOS>	<PAD>			

#### LSTM Translation Model

**Figure 5:**
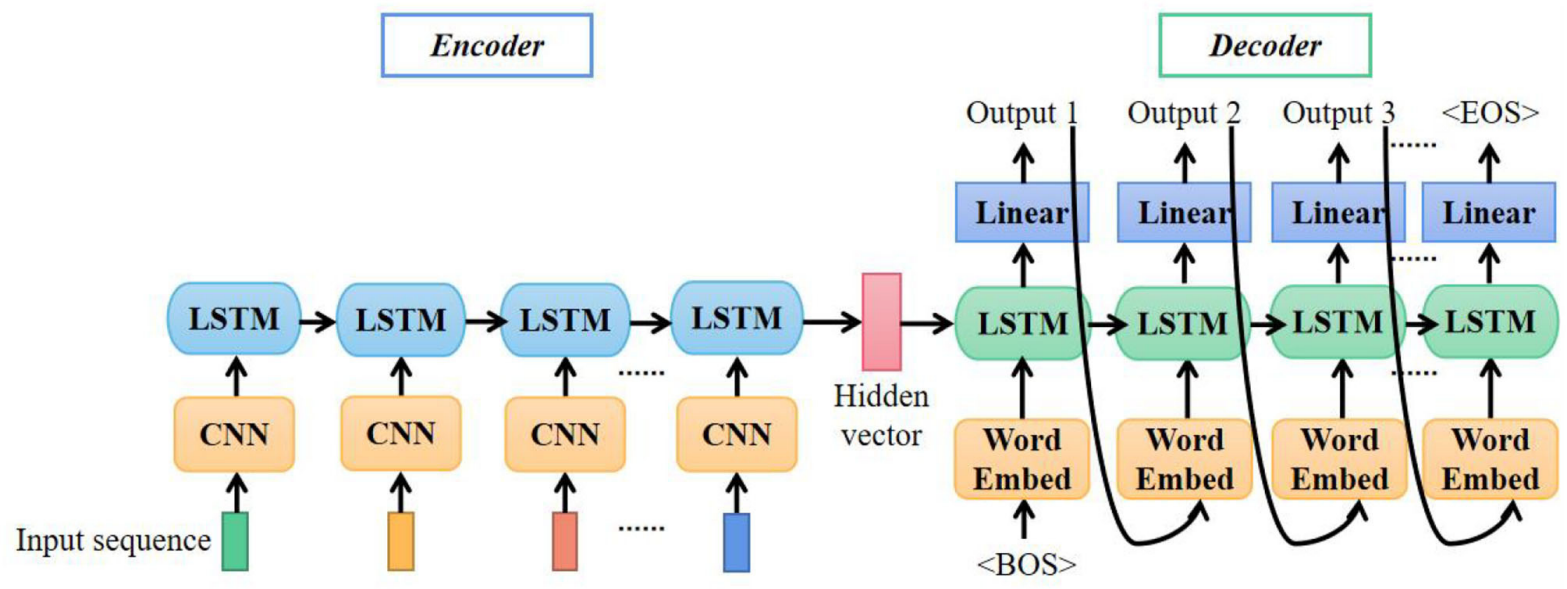
Architecture of long short-term memory (LSTM)-based translation model.

The first model is based on LSTM. As illustrated in Figure 5, the first layer of the encoder is CNN. The CNN layer extracts superior representations of features from input data frames as introduced in Section Facial Expressions Classifier. The input signal of stacked IMU and EMG had 40 channels, so the convolutional kernels we used here were 2-D kernels with the shape of 3 × 3.

**Figure 6:**
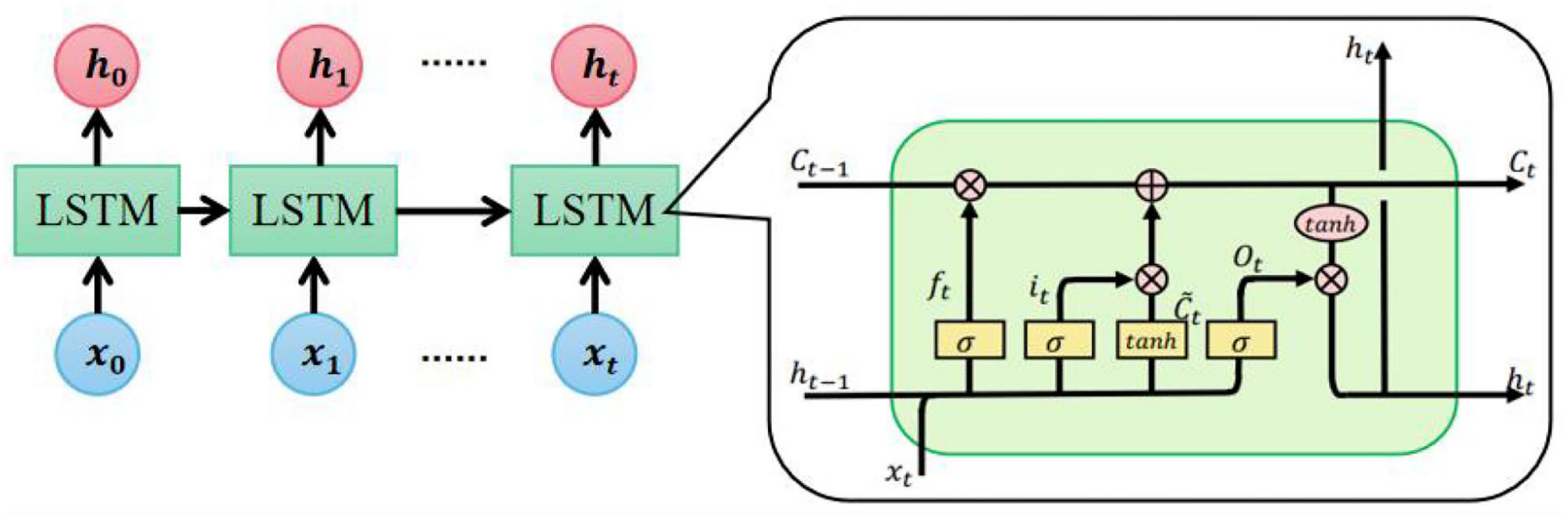
Detailed structure of long short-term memory (LSTM) unit.

The second layer of the encoder is LSTM. LSTM is widely used in speech recognition, language modeling, and translation to model temporal dependence. As an extended model of Recurrent Neural Network (RNN), LSTM can preserve the long-term dependencies by controlling the percentage of previous information dropping, current information inputting, and current information outputting (Shi et al., 2015). Figure 6 shows the LSTM expanded by time step and the detailed structure of the LSTM unit.

The cell state *C*_*t*−1_ and hidden state *h*_*t*−1_ from the previous time step along with the current input *x*_*t*_ are the inputs to the current LSTM unit. The forget gate *f*_*t*_, input gate *i*_*t*_, update gate $\tilde{C_{t}}$, and output *o*_*t*_ are calculated as follows:


$$\begin{array}{l} f_{t}\;=\;\sigma \left(W_{f}\cdot \left[h_{t-1},\;x_{t}\right]+b_{f}\right)\\ i_{t}\;=\;\sigma \left(W_{i}\cdot \left[h_{t-1},\;x_{t}\right]+b_{i}\right)\\ \tilde{C_{t}}\;=\;\tanh\left(W_{C}\cdot \left[h_{t-1},\;x_{t}\right]+b_{c}\right)\\ o_{t}\;=\;\sigma \left(W_{o}\cdot \left[h_{t-1},\;x_{t}\right]+b_{o}\right) \end{array}$$

Where σ is the sigmoid function, and *W are b* are weights and bias, respectively. With these results, *C*_*t*_ and *h*_*t*_ are updated:


$$\begin{array}{l} C_{t}\;=\;f_{t}{\;}^{*}C_{t-1}\;+\;i_{t}{\;}^{*}\tilde{C_{t}}\\ h_{t}\;=\;o_{t}{\;}^{*}\tanh\left(C_{t}\;\right) \end{array}$$

The hidden vectors *C*_*t*_ and *h*_*t*_ passed to the decoder were used as the initial hidden state of decoder LSTM. Given the special word <BOS>, the decoder started to output predicting results step by step. If the output of a time step was turned to <EOS>, the whole predicting procedure should be finished.

#### Transformer Translation Model

**Figure 7:**
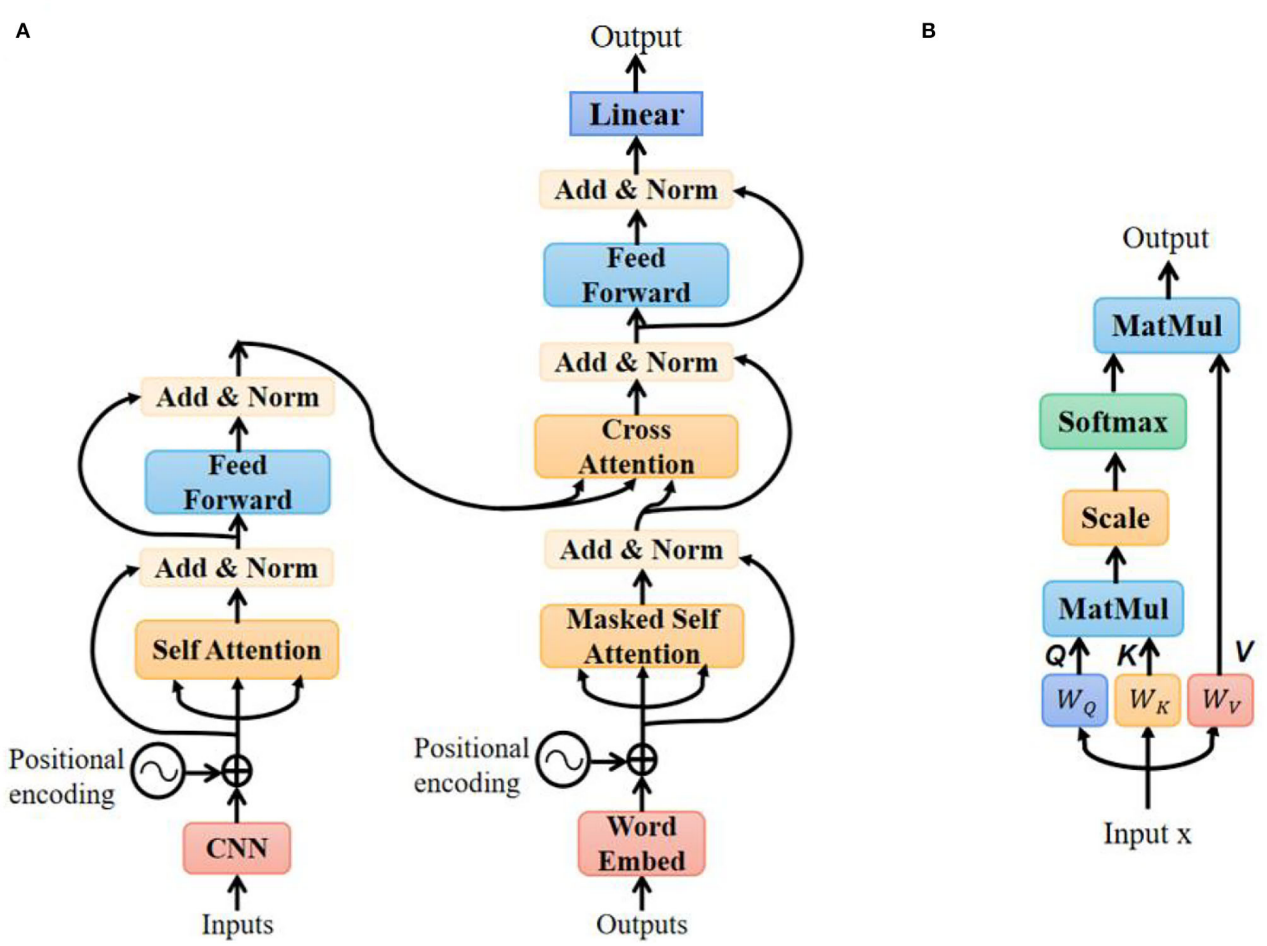
Transformer-based translation model: **(A)** architecture of the model; **(B)** detailed structure of the self-attention layer.

The transformer model has been used successfully in a variety of tasks, such as reading comprehension, textual entailment, and learning task-independent sentence representations (Vaswani et al., 2017). With the self-attention mechanism, the model can draw global dependencies between input and output without considering the distance. The architecture of the transformer-based translation model is shown in Figure 7A.

In the encoder section, the input to the self-attention layer consists of two parts: features' sequence extracted from the CNN layer and positional encoding recording the sequence order. The detailed structure of the self-attention layer is shown in Figure 7B. Query, key, and value all come from the same input by performing different linear transformations:


$$\begin{array}{l} Q\;=\;W_{Q}\;\cdot \;x\\ K\;=\;W_{K}\;\cdot \;x\\ V\;=\;W_{V}\;\cdot \;x \end{array}$$

The attention score is calculated as:


$$\begin{array}{l} scores\;=\;softmax\left(\frac{Q\cdot K^{T}}{\sqrt{d_{k}}}\right) \end{array}$$

Where *d*_*k*_ is the dimension of *K*. The output of the self-attention layer is matrix multiplication between score and value matrix *V*:


$$\begin{array}{l} Attention\left(Q,\;K,\;V\right)\;=\;scores\;\cdot \;V \end{array}$$

After going through layer normalization and feed-forward module, the input was finally encoded into a hidden vector.

In the model's training step, the input of the decoder was a text sentence. In the masked self-attention layer, the model could only attend to the output words that had been predicted before. The encoder-decoder cross-attention layer includes *K* and *V* from encoder output and *Q* from decoder input. The calculation method is the same as self-attention. The output of the decoder is the probabilities of all possible words in the vocabulary. With the greedy search decoding method (Edmonds, 1971), we chose the word with the largest probability as the model prediction.

## Results

### Facial Expressions Classification

We validated the CNN classifier with 5-fold cross-validation. The dataset of EMG signals that contained 2,400 samples was randomly divided into five subsets. We left each subset as the validation set and trained the model with the remaining four subsets. This process was repeated five times. The loss function of the model was cross-entropy loss and the optimizer was Adam with a learning rate of 0.001. According to the recognition results of validation sets, the classification accuracy is calculated as given below:


$$\begin{array}{l} Accuracy\;=\;\frac{Number\;of\;correct\;classifications}{Total\;number\;of\;validation\;samples} \end{array}$$

**Figure 8:**
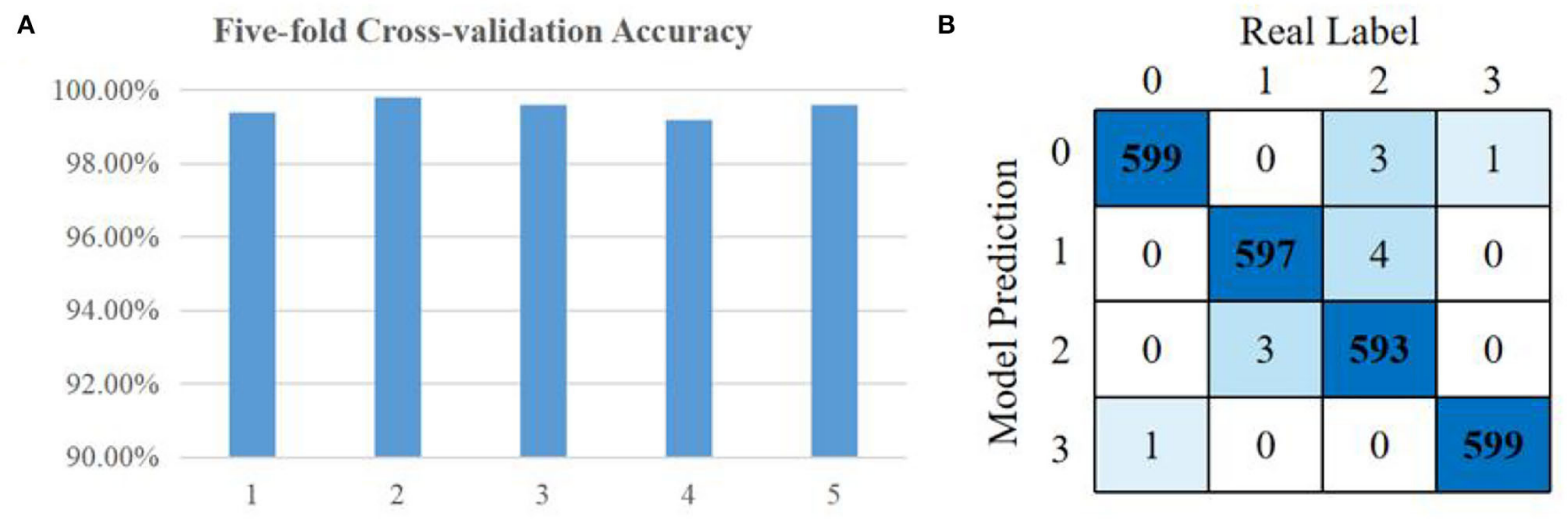
Facial expressions classification results: **(A)** accuracy of five cross-validation sets; **(B)** total confusion matrix of cross-validation steps.

After training the model, the classification results of all cross-validation sets are shown in Figure 8A. The accuracy of more than 99% illustrates that EMG features are significantly different in four kinds of facial expressions. The confusion matrix accumulated from all cross-validation steps is shown in Figure 8B.

### Sign Language Translation

We randomly divided 2,400 samples in the dataset into a training set (70%, 1,680 samples), validation set (15%, 360 samples), and testing set (15%, 360 samples). We used data in the training set to train the model and then adjusted parameters with the validation set to select the model with the best performance. The training loss is cross-entropy between model prediction and real labeled sentences. The optimizer is Adam with a learning rate of 0.0003.

On the testing set, we employed word error rate (WER) and sentence error rate (SER) as the evaluation of the model. WER measures the least operations of substitution, deletion, and insertion to transform the predicted sentence into the ground truth sentence:


$$\begin{array}{l} WER\;=\;\frac{N_{sub}\;+\;N_{del}\;+\;N_{ins}}{N_{ground\;truth\;words}} \end{array}$$

Where *N*_*sub*_, *N*_*del*_, and *N*_*ins*_ are numbers of required substitutions, deletions, and insertions, respectively. SER measures the percentage of not completely correct sentences of the model's testing prediction results:


$$\begin{array}{l} SER\;=\;\frac{N_{error\;sentences}}{N_{ground\;truth\;sentences}} \end{array}$$

**Figure 9:**
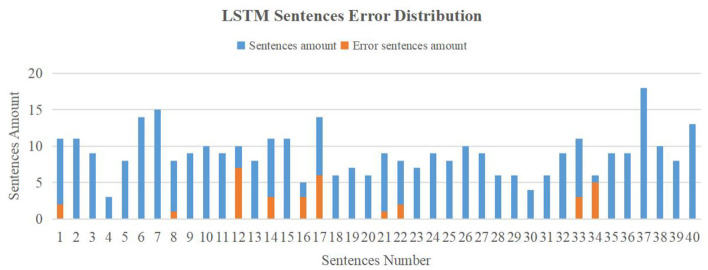
Long short-term memory (LSTM) model evaluation result.

In the training step of the LSTM translation model, the losses of training and validation set were both dropped dramatically in the first few epochs. After 15 epochs of training, the model tended to converge with a loss of nearly 0. We stopped training the model at epoch 20 and evaluated it with the testing set. Figure 9 shows the evaluation result of the LSTM translation model on the testing dataset. The blue bars are the sentence amount distribution of 40 sign language sentences in the testing set and the orange bars show the error sentences amount. Most sentences were predicted correctly by the model. The SER we calculated was 9.17% (33 error sentences of 360 samples) and the WER was 7.74% (43 del errors, 17 ins errors, and 87 sub errors of 1,898 words).

**Figure 10:**
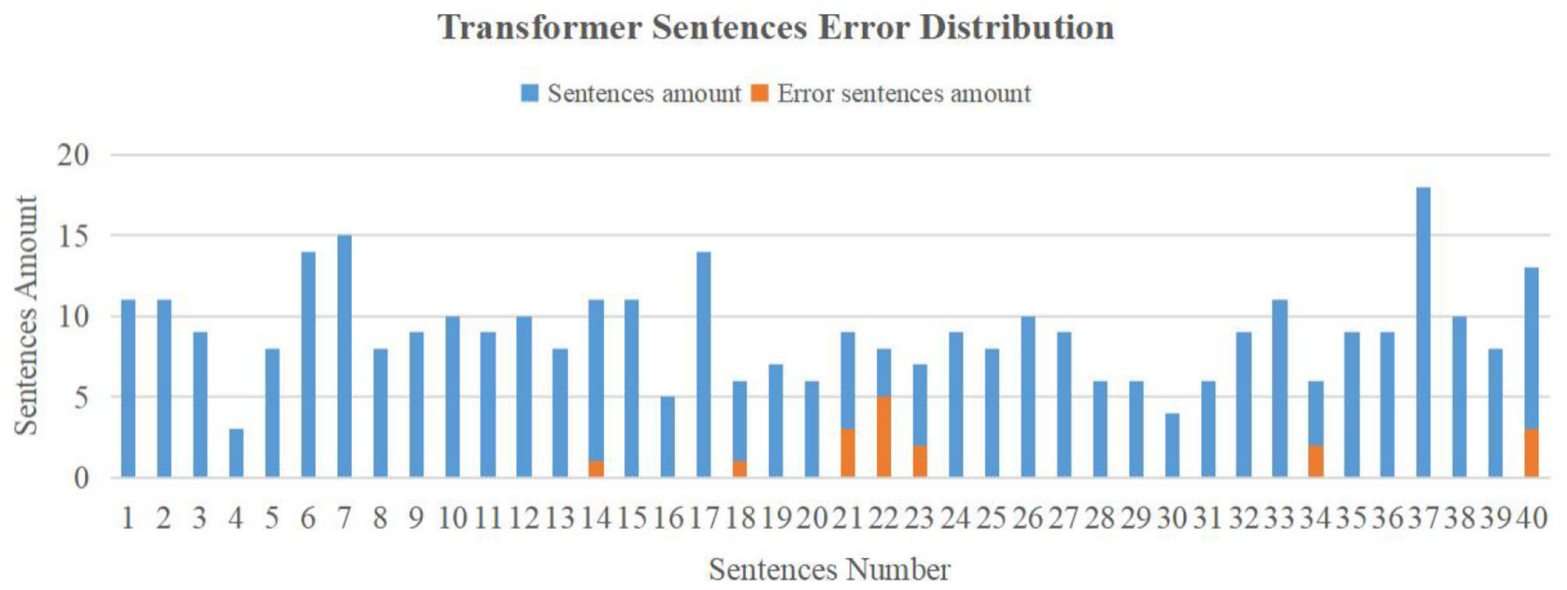
Transformer model evaluation result.

The transformer translation model converges much faster, so we trained the model for only 15 epochs. The evaluation result is shown in Figure 10. This model performs much better than the LSTM model in the testing dataset. There were only 17 error sentences from 360 sentences in the dataset and thus the SER was 4.72%. The WER was calculated to be 4.21% (33 del errors and 47 sub errors of 1,898 words).

## Discussion

### Significance of EMG

In this work, EMG signals from facial areas provided four kinds of emotional information during sign language performance. Combining EMG and IMU data as input provides the model with more information to achieve better prediction results. To evaluate the significance of EMG, we removed the EMG data from the input and then trained the translation models again with only IMU data.

The comparisons between input with or without EMG are shown in Tables 3, 4. WERs of the two models increase by 4.12 and 4.21% without EMG data as input, and SERs also increase by 5.55 and 4.17%, respectively. Both models gave more wrong predictions, but the transformer model still performed much better than the LSTM model at a 3.43% lower error rate at the word level and 5.83% lower error rate at the sentence level.

**Table 3:** Word error rate comparison.

	**LSTM**	**Transformer**
Input with EMG	7.74%	4.22%
Input without EMG	11.86%	8.43%

**Table 4:** Sentence error rate comparison.

	**LSTM**	**Transformer**
Input with EMG	9.17%	4.72%
Input without EMG	14.72%	8.89%

### User-Independent Validation

We evaluated the performance of models in user-independent conditions. Three participants participated in this experiment. Participant 1 who contributed the largest amount of data (1,600 samples) was always used as a part of the training set. Participants 2 (400 samples) and 3 (400 samples) were regarded as testing sets, respectively. The results are shown in Table 5. In the sign language translation task, both WER and SER increased dramatically to more than 40%. Due to different habits and amplitudes of each person's sign language performances, there were great differences between the movement data in user-independent validation. The method we proposed could still translate more than half of the sentences in the testing set accurately. In the user-independent validation of facial expression classification with EMG, the accuracy remained at a high level of more than 93%. The result illustrated that the EMG signals of four different expressions had distinguishable features.

**Table 5:** User-independent validation results.

	**WER**	**SER**	**Facial expression classification accuracy**
Participant 2	41.95%	44.50%	93.25%
Participant 3	41.12%	46.00%	95.00%

### Limitations

The dataset contains limited sentences and participants. Only four kinds of facial expressions were considered, as a result, the CNN classifier gave high-accurate results on this four-category classification task. LSTM and transformer are two commonly used models in NLP research. Instead of text or speech, the input of sign language is signals from the human body. The transformer model outperforms the LSTM model. The transformer is originally proposed to solve the sequential order problem of RNN (Vaswani et al., 2017). The LSTM model can only read input from left to right or from right to left, but the transformer considers the overall input content at the same time. With EMG as a part of the input, the accuracy of the model prediction improves. EMG can enhance the model's translation ability. In user-independent validation, the translation accuracy dropped dramatically due to the significant inter-individual differences in movement. More participants should be involved in the experiment and the model should learn knowledge from more data.

Compared with visual methods of sign language translation, a camera is more portable but will encounter background and perspective problems. Even the most popular Kinect camera with skeleton tracking function cannot extract the detailed skeleton structure of hands. To some extent, wearable IMU sensors are more reliable. The IMU-based motion capture device for the upper body contains 25 sensors. It is a unitary device and cannot be disassembled. This motion capture system is bulky for a translation system with only 40 sentences, but it has the potential to recognize more sentences. A larger dataset using this device is in preparation and machine-learning algorithms more suitable for wearables are being developed.

## Conclusion

In this paper, we presented a wearable sensor-based sign language translation method considering both hands' movements and facial expressions. IMU and EMG signals were preprocessed and segmented into a sequence of frames as the input of translation models. We classified facial expressions with EMG data only. Then we built encoder-decoder models to realize end-to-end sign language translation from signals to text sentences. Two kinds of end-to-end models based on LSTM and transformer were trained and evaluated by the collected dataset. WER and SER were used to compare the translation ability of models. Both models could translate 40 ASL sentences with high accuracy and the transformer-based model performed better than LSTM. The special role of EMG was verified with both facial expressions' classification and models' performance after removing EMG from the input. The translation accuracy in user-independent conditions was evaluated.

## Data Availability Statement

The raw data supporting the conclusions of this article will be made available by the authors, without undue reservation.

## Ethics Statement

Ethical review and approval was not required for the study on human participants in accordance with the local legislation and institutional requirements. The patients/participants provided their written informed consent to participate in this study.

## Author Contributions

YG: conceptualization, methodology, software, validation, data curation, and original draft preparation. CZ: methodology, formal analysis, validation, review, and editing. MT: methodology, review and editing, project administration, and funding acquisition. FZ: conceptualization, methodology, review, and editing. All authors have read and agreed to the published version of the manuscript.

## Funding

This research was funded by Hokkaido University DX Doctoral Fellowship, grant number JPMJSP2119.

## Conflict of Interest

CZ was employed by company China State Shipbuilding Corporation Limited. The remaining authors declare that the research was conducted in the absence of any commercial or financial relationships that could be construed as a potential conflict of interest.

## Publisher's Note

All claims expressed in this article are solely those of the authors and do not necessarily represent those of their affiliated organizations, or those of the publisher, the editors and the reviewers. Any product that may be evaluated in this article, or claim that may be made by its manufacturer, is not guaranteed or endorsed by the publisher.
